# Chloral hydrate enteral infusion for sedation in ventilated children: the CHOSEN pilot study

**DOI:** 10.1186/s13054-017-1879-7

**Published:** 2017-11-26

**Authors:** Ari R. Joffe, Jessica Hogan, Cathy Sheppard, Gerda Tawfik, Jonathan P. Duff, Gonzalo Garcia Guerra

**Affiliations:** 1 Department of Pediatrics, Division of Pediatric Critical Care Medicine, Stollery Children’s Hospital and University of Alberta, 8440 112 Street, Edmonton, Alberta T6G 2B7 Canada; 2 Department of Nursing, Division of Pediatric Critical Care Medicine, Stollery Children’s Hospital and University of Alberta, 8440 112 Street, Edmonton, Alberta T6G 2B7 Canada; 3 Department of Pharmacy, Division of Pediatric Critical Care Medicine, Stollery Children’s Hospital and University of Alberta, 8440 112 Street, Edmonton, Alberta T6G 2B7 Canada; 4 4-546 Edmonton Clinic Health Academy, 11405 87 Avenue, Edmonton, Alberta T6G 1C9 Canada

**Keywords:** Chloral hydrate, Intensive care units, Pediatric, Mechanical ventilation, Moderate sedation

## Abstract

**Background:**

We aimed to test a novel method of delivery of chloral hydrate (CH) sedation in ventilated critically ill young children.

**Methods:**

Children < 12 years old, within 72 hours of admission, who were ventilated, receiving enteral tube-feeds, with intermittent CH ordered were enrolled after signed consent. Patients received a CH loading-dose of 10 mg/kg enterally, then a syringe-pump enteral infusion at 5 mg/kg/hour, increasing to a maximum of 9 mg/kg/hour. Cases were compared to historical controls matched for age group and Pediatric Risk of Mortality score (PRISM) category, using Fisher’s exact test and the *t* test. The primary outcome was feasibility, defined as the use of an enteral CH continuous infusion without discontinuation attributable to a pre-specified potential harm.

**Results:**

There were 21 patients enrolled, at age 11.4 (12.1) months, with bronchiolitis in 10 (48%), a mean Pediatric Logistic Organ Dysfunction (PELOD) score of 6.2 (5.2), and having received enteral CH continuous infusion for 4.5 (2.2) days. Infusion of CH was feasible in 20/21 (95%; 95% CI 76–99%) patients, with one (5%) adverse event of duodenal ulcer perforation on day 3 in a patient with croup receiving regular ibuprofen and dexamethasone. The CH infusion dose (mg/kg/h) on day 2 (*n* = 20) was 8.9 (IQR 5.9, 9), and on day 4 (*n* = 11) was 8.8 (IQR 7, 9). Days to titration of adequate sedation (defined as ≤ 3 PRN doses/shift) was 1 (IQR 0.5, 2.5), and hours to awakening for extubation was 5 (IQR 2, 9). Cases (versus controls) had less positive fluid balance at 48 h (-2 (45) vs. 26 (46) ml/kg, *p* = 0.051), and a decrease in number of PRN sedation doses from 12 h pre to 12 hours post starting CH (4.7 (3.3) to 2.6 (2.8), *p* = 0.009 versus 2.9 (3.9) to 3.4 (5), *p* = 0.74). There were no statistically significant differences between cases and controls in inotrope scores, signs or treatment of withdrawal, or PICU days.

**Conclusions:**

Delivering CH by continuous enteral infusion is feasible, effective, and may be associated with less positive fluid balance. Whether there is a risk of duodenal perforation requires further study.

**Electronic supplementary material:**

The online version of this article (doi:10.1186/s13054-017-1879-7) contains supplementary material, which is available to authorized users.

## Background

Sedation in the pediatric intensive care unit (PICU) is an essential part of management of the critically ill child receiving mechanical ventilation. Sedation facilitates safe therapeutic and diagnostic procedures, ensures patient comfort, reduces distress in the child and as a result also reduces parental anxiety [1, 2]. Ensuring adequate sedation in the PICU is often a challenge, requiring multiple drugs, delaying extubation, and resulting in drug withdrawal and delirium [1–5]. Over-sedation is associated with hypotension, fluid administration, and inability to assess underlying neurological status, any of which can delay extubation and PICU discharge [1–3]. Conversely, under-sedation is associated with anxiety, fear, and discomfort, and can lead to losing vascular lines and invasive airways [1, 2]. A recent consensus guideline on sedation and analgesia in critically ill children recommended the early use of enteral sedative agents [6].

Chloral hydrate (CH) is an enteral sedative/hypnotic drug that has been used for decades for sedation in children [7, 8]. It is common practice to administer CH in repetitive doses to maintain prolonged sedation in children during mechanical ventilation [6, 9–12]. A survey on the use of sedatives in the PICU in the USA found that 45% of intensivists used CH frequently or routinely; > 60% responded that they used CH for long-term sedation [9]. A survey of Canadian pediatric intensivists found that CH was among the most frequently used sedative medications [12]. A previous study has shown the greater efficacy of oral sedatives such as CH compared to midazolam, a sedative frequently given in the PICU [13]. A recent study including 343 PICU patients who received CH for a median of 6 days at a dose of 134.4 (SD 83.2) mg/kg/day found that CH was associated with similar rates of adverse reactions (mainly desaturation and hypotension) as other sedatives used in the PICU [14].

Our anecdotal observation was that patients often are initially over-sedated following a dose of CH, but are then under-sedated before the next dose is due; a continuous enteral infusion of CH may blunt this fluctuation in sedation level, with a lower total dose and fewer adverse effects. In addition, continuous enteral infusion of CH may result in dose-sparing of other sedatives that have a higher potential for adverse effects, delayed awakening, and withdrawal, including benzodiazepines and opioids [1–4]. We hypothesized that a novel, simple, and low-cost method of delivering enteral CH, continuous enteral infusion, is feasible as a sedative infusion in critically ill children. We also hypothesized that continuous enteral infusion of CH is associated with important patient outcomes including lower opioid and benzodiazepine requirements, and improved outcomes in fluid balance, ventilator days, inotropic support, and harms in the PICU.

## Methods

### Ethics approval

This study was approved by the Health Research Ethics Board of the University of Alberta (Pro00035544). All case patients had their parent or legal guardian sign informed consent to participation.

### Study design

This is a prospective cohort study of enteral infusion of CH. Eligibility criteria were the following: admitted to the PICU at Stollery Children’s Hospital; PICU admission for < 72 h; mechanically ventilated; ≥ 44 weeks post-conceptional age up to 12 years of age; CH sedation ordered by the treating PICU team; and enteral feeding tube delivering feeds at any rate by nasogastric, nasojejunal, gastric, or gastrojejunal tube. Exclusion criteria were any of the following: weight <3 kg; contraindication to the use of the gastrointestinal tract to receive oral medication; short gut syndrome; known gastric or duodenal ulcer; severe liver dysfunction defined by INR >2 and lactate >2 mmol/L; severe renal dysfunction (defined by needing dialysis); or allergy to CH.

The intervention patients (CHOSEN cohort) were compared to a matched historical control group (control cohort) of patients that received intermittent CH in the year prior to the intervention. These historical controls were matched for age (<1 year or 1–5 years) and Pediatric Risk of Mortality (PRISM) score (<10 or 10–20) to intervention patients. In the historical controls, baseline was considered the time that CH was ordered by the attending physician. The historical controls met the same eligibility criteria, and were identified retrospectively through a pharmacy database by determining patients who had CH removed from the on-site PICU computerized dispensing system. Because many patients who had CH sedation in the PICU were not tracked through this system, to have enough patients to match, these controls were from periods both before and after the CHOSEN cohort was completed.

### Study procedures

A CHOSEN order sheet with instructions on dosing and using the infusion pump (for safety, programmed with hard dosing limits, and to require confirmation that infusion was given enterally) was used (Additional file 1). CHOSEN patients were to receive a loading dose of CH 10 mg/kg enterally, followed by a CH enteral infusion by syringe pump at 5 mg/kg/h Y-infused into the feeding tube with the feeds. The CH infusion could be increased up to a maximum of 9 mg/kg/h enterally. As there was no published information on continuous CH dosing, this dosing algorithm was based on our experience that intermittent CH in our PICU was usually ordered at doses of 15–30 mg/kg every 3 hours as required (prn). Each increment in the enteral CH infusion was by 1 mg/kg/h following a loading dose of 3 mg/kg, and could occur no more frequently than hourly. If the patient was over-sedated for at least 4 h without any prn medications, the other sedation medications being used were to be decreased at the attending medical team’s discretion prior to considering decreasing the CH infusion by 1–2 mg/kg/h up to every 3 h. When decreasing CH infusion in anticipation of extubation, the instruction was to decrease the CH infusion rate by half at 6 h prior to extubation, and to stop the CH infusion 3 h prior to extubation. Prior to the study, we tested an in vitro infusion of CH mixed with commonly used PICU feeding formulas infused at low rates to maximize the chance of tube blockage, and found no tube blockage after more than 24 h.

Clinical data were recorded from the patient chart onto a case report form with pre-specified definitions for all variables (Additional file 2). Baseline variables included demographics (age, sex, weight, diagnostic category, surgery category), and severity of illness measures (inotrope score, PRISM score, and Pediatric Logistic Organ Dysfunction (PELOD) score) [15–17]. Outcome variables from baseline included: sedation needs (rescue prn sedation doses per day for days 1–7; time to titration of adequate sedation defined as ≤ 3 prn sedation doses per 12-h shift; total daily dose (prn and infusion) of CH, benzodiazepine, opioid, and dexmedetomidine for days 1–7); time to awakening on discontinuation or lowering of sedation infusions in preparation for extubation, defined as awake enough for extubation to occur; fluid balance at 24 h and 48 h, in ml/kg; ventilator hours; and PICU length of stay in days. Potential harms were pre-specified to include: feeding tube blockage requiring tube replacement; gastrointestinal bleeding requiring transfusion of at least 10 ml/kg packed red blood cells; new or worsened seizures treated with anticonvulsant; new or worsened ventricular dysrhythmia requiring treatment; feed intolerance defined as feeds being held for > 3 h; or failed extubation due to excessive remaining sedative effect (e.g., hypoventilation, or excessive secretions).

### Statistics

There are no previous studies of CH enteral infusion. The primary outcome is feasibility, defined as the use of an enteral CH continuous infusion without discontinuation attributable to a pre-specified potential harm (see above for definition) or lack of adequate sedation. Assuming that CH infusion would be feasible in at least 75% of CHOSEN patients, we estimated that a sample size of 32 patients would be needed to estimate the feasibility within 15% of the true feasibility rate with 95% confidence. This was calculated as follows:

$$ n={\left(1.96/0.15\right)}^2\uppi \left(1-\uppi \right), $$

where *n* is the number of patients needed, π is the proportion of patients in whom the CH infusion is expected to be feasible (=0.75), and 0.15 is the maximum discrepancy between the study sample feasibility and the population feasibility with a certainty of 95%. The main pre-specified secondary outcomes are: number of prn doses of sedation per day and their change from pre CH to post CH, time to titration to adequate sedative effect, time to awakening on discontinuation or lowering of sedation infusions, and fluid balance at 48 h. Descriptive summary measures including mean (standard deviation, SD) and median (interquartile range, IQR) were used as appropriate. The Student *t* test and chi-square analysis, as appropriate, were used to explore the differences between and within groups (enteral CH infusion vs. historical controls) in the secondary outcomes.

## Results

### Description of cohorts

From June 2013 to May 2014 there were 140 patients screened and 30 patients eligible for the study; 7 patients were not approached (1 whose family did not speak English, 1 who was apprehended by social services, and 5 who were not approached because there were no research staff available), 2 patients declined consent, and 21 patients were prospectively enrolled into the CHOSEN group. The historical controls were retrospectively identified patients from February to December 2012 and August 2014 to May 2016. Most baseline variables for CHOSEN cases and historical controls were similar, except that the controls included cardiac surgical patients (5 (24%) vs. 0%, *p* = 0.048), some patients that had non-invasive mechanical ventilation (3 (14%) vs. 0%, *p* = 0.23), and fewer patients with the diagnosis of bronchiolitis (4 (19%) vs. 10 (48%), *p* = 0.10) and with medical illness (13 (62%) vs. 18 (86%), *p* = 0.16) (Table 1). All patients were < 6 years old, mostly infants, and with moderate severity of illness as measured by inotrope score, PRISM, and PELOD scores (Table 1).

**Table 1:** Baseline variables for cases and controls

Variable	CHOSEN (*n* = 21)	Controls (*n* = 21)	*p* value
Age (months)	11.4 (12.1)	14.0 (19.6)	0.61
	7 (IQR 3, 18)	9 (IQR 5, 13)	
Age category (<1; 1–5; 6–12 years)	13; 8; 0	16; 4; 1	0.27
Weight (kg)	8.6 (3.6)	8.3 (5.5)	0.87
	8.9 (IQR 5.5, 11.2)	7 (IQR 4.6, 9.8)	
Gender male	15 (71%)	11 (52%)	0.34
Cardiac surgical	0	5 (24%)^a^	0.048
General surgical	4 (19%)	5 (24%)	0.99
Medical	18 (86%)	13 (62%)	0.16
Bronchiolitis	10 (48%)	4 (19%)	0.10
Inotrope used	2 (10%)	2 (10%)	0.99
Epinephrine or norepinephrine used	2 (10%)	2 (10%)	0.99
Inotrope score	0.5 (1.5)	1.1 (4.4)	0.52
Absolute inotrope scores	5, 5	4, 20	
Lactate measured	19	21	0.49
Lactate (mmol/L)	0.7 (0.2)	1.0 (0.6)	0.02
Creatinine measured	15	17	0.72
Creatinine (umol/L)	21 (5)	21 (10)	0.81
PRISM	2.5 (2.3)	3.0 (2.5)	0.48
	3 (0, 5)	3 (1, 5)	
PELOD	6.2 (5.2)	6.5 (5.0)	0.88
	2 (1, 11)	10 (1, 11)	
Invasive ventilation	21 (100%)	18 (86%)^b^	0.23

Data given as *n* (%), or mean (SD), or median (IQR). Comparisons by Fisher’s exact test and t test, as appropriate. *PELOD* pediatric logistic organ dysfunction score, *PRISM* pediatric risk of mortality score

^a^congenital heart disease were: atrial septal defect (*n* = 1); tetralogy of Fallot (*n* = 2); other (*n* = 1)

^b^Three control patients had only non-invasive ventilation used

### Primary outcome

The CH enteral infusion was feasible in all 21 (100%) CHOSEN patients using the original definition of feasibility: use of an enteral CH continuous infusion without discontinuation attributable to a pre-specified potential harm or lack of adequate sedation. Patients were on CH enteral infusion for 4.5 (SD 2.2) days, and 4.4 (IQR 2.3, 6.7) days (Table 2); the highest infusion dose on day 1 and 2 was 6.4 (SD 1.0) and 7.4 (SD 2.3) mg/kg/h (Table 3). There was one serious adverse event in a 16-month-old girl with croup due to parainfluenza virus, who was intubated and ventilated and treated with dexamethasone every 6 h (q6h) and ibuprofen q6h for 3 days prior to developing a perforated duodenal ulcer that required laparotomy and Graham patch. This event was thought possibly related to the enteral CH infusion by the medical team; as this was a potential harm, the CH enteral infusion was feasible in 20/21 (95%; 95% adjusted Wald CI 76–99%). There was one death among patients in the historical control group (5%) and no deaths in the CHOSEN group.

**Table 2:** Outcomes in cases and controls

Outcome	CHOSEN (*n* = 21)	Controls (*n* = 21)	*p* value
Days on study			
	4.5 (2.2)	-	-
	4.4 (IQR 2.3, 6.7)		
Primary outcomes			
Pre-defined feasibility	21/21 (100%)	-	-
Any pre-specified adverse effect^a^	0	0	-
SAE^b^	1 (5%)	0	0.99
Mortality in PICU	0	1 (5%)	0.99
Post-hoc feasiblity	20/21 (95%)	20/21 (95%)	0.99
Main secondary outcomes			
Days to titration of adequate sedation	1.4 (1.3)	0.9 (1.3)	0.22
	1 (IQR 0.5, 2.5)	0.5 (IQR 0, 1.3)	
Hours to awakening	N = 19	N = 18	0.80
	8.1 (8.4)	8.8 (8.5)	
	5 (IQR 2, 9)	8 (IQR 1, 13)	
Fluid balance, 48 h (ml/kg)	−2 (45)	26 (46)	0.051
	−7 (IQR −30, 25)	19 (IQR −4, 59)	
Number of prn sedation doses from 12 h pre to 12 h post baseline	4.7 (3.3) to 2.6 (2.8)	2.9 (3.9) to 3.4 (5.0)	0.009/0.74
Other secondary outcomes			
Ventilator hours	125 (84)	258 (363)	0.12
	106 (IQR 54, 162)	129 (IQR 63, 191)	
PICU days	9.8 (7.6)	16.8 (21.7)	0.17
	8.5 (IQR 5, 13)	8 (IQR 5.5, 12)	
Extubation information			
Propofol used as bridge to extubation	13 (62%)	6 (29%)	0.06
Failed extubation due to sedation	0/21	0/19	-
Post-extubation withdrawal syndrome			
Signs of withdrawal	9/20 (45%)	8/19 (42%)	0.99
Enteral narcotic started within 48 h	3 (15%)	4 (22%)	0.99
Enteral BDZ started within 48 h	4 (19%)	2 (11%)	0.66
Enteral clonidine started within 48 h	3 (15%)	2 (11%)	0.99
Withdrawal score 48 h after extubation	N = 12/21 (57%)	N = 14/21 (67%)	0.53
	4.3 (3.3)	2.3 (2.5)	0.10
Fluid balance, 24 h (ml/kg)	5 (32)	24 (31)	0.06
	3 (IQR −17, 32)	21 (IQR −3, 49)	

Data given as *n* (%), mean (SD), or median (IQR). Comparisons by Fisher’s exact test and *t* test or paired *t* test, as appropriate. *PICU* pediatric intensive care unit, *BDZ* benzodiazepine, *SAE* serious adverse event, *prn* as required

^a^Pre-specified adverse effects were defined as any of: feeding tube blockage, GI bleeding, new/worse seizure, feed intolerance, or new/worse ventricular dysrhythmia

^b^Chloral hydrate infusion was feasible on day 1 to 7, except for one patient on day 3 due to the SAE (duodenal perforation; see text)

**Table 3:** Sedation used during the 7 days of study in patient cases and controls

Variable	Group	Day 1	Day 2	Day 3	Day 4	Day 5	Day 6	Day 7
Doses of sedation given prn (excluding chloral hydrate) (number of doses (SD))								
Total	CHOSEN	*N* = 21 (12 h)	*N* = 20	*N* = 17	*N* = 12	*N* = 8	*N* = 7	*N* = 5
		2.6 (2.8)	3.5 (3.5)	3.5 (4.7)	2.6 (2.2)	4.5 (3.3)	4.7 (4.8)	4.8 (3.9)
	Control	*N* = 21 (12 h)	*N* = 20	*N* = 14	*N* = 10	*N* = 6	*N* = 4	*N* = 3
		3.4 (5.0)	2.4 (2.9)	2.4 (3.5)	3.0 (3.1)	1.8 (1.7)	2.3 (1.5)	1.0 (1.0)
	*p* value	0.54	0.29	0.45	0.72	0.10	0.35	0.16
Description of chloral hydrate dosing (mg/kg/day; and mg/kg/h infusion)								
Total chloral dose given	CHOSEN	*N* = 21	*N* = 20	*N* = 17	*N* = 11	*N* = 8	*N* = 7	*N* = 5
		116 (50)	160 (50)	138 (84)	140 (81)	152 (74)	143 (91)	134 (86)
	Control	*N* = 21	*N* = 20	*N* = 14	*N* = 10	*N* = 6	*N* = 4	*N* = 3
		38 (34)	42 (49)	37 (34)	75 (117)	64 (43)	40 (32)	33 (29)
	*p* value	<0.001	<0.001	<0.001	0.15	0.02	0.03	0.06
Highest chloral infusion rate	CHOSEN	6.4 (1.0)	7.4 (2.3)	7.2 (2.1)	7.7 (2.0)	6.6 (2.7)	6.7 (3.3)	6.4 (2.7)
		7 (IQR 5, 9)	8.9 (IQR 5.9, 9)	8 (IQR 5.6, 9)	8.8 (IQR 7, 9)	7.1 (IQR 4, 9)	8.8 (IQR 3.3, 9)	5.2 (IQR 2.5, 9)
Inotrope requirements while on chloral hydrate (inotrope score (SD))								
Highest inotrope score	CHOSEN	*N* = 21	*N* = 20	*N* = 17	*N* = 12	*N* = 8	*N* = 7	*N* = 5
		0.5 (1.5)	0.9 (2.3)	1.1 (3.3)	1.3 (3.7)	1.8 (3.9)	1.1 (3.0)	1.0 (2.2)
	Control	*N* = 21	*N* = 20	*N* = 14	*N* = 11	*N* = 6	*N* = 4	*N* = 3
		1.5 (4.6)	0.7 (1.7)	0.6 (1.6)	0.9 (3.0)	1.0 (2.4)	1.0 (2.0)	0 (0)
	*p* value	0.37	0.76	0.67	0.75	0.69	0.94	0.48
On inotropes (absolute score)	CHOSEN	3/21 (14%)	3/20(15%)	2/17(12%)	2/12(17%)	2/8(25%)	1/7 (14%)	1/5(20%)
		1,5,5,	5,5,8	5,13	5,11	3,11	8	5
	Control	3/21 (14%)	3/20 (15%)	2/14 (14%)	1/11 (9%)	1/6 (17%)	1/4 (25%)	0
		3, 8, 20	4, 5, 5	4, 5	10	6	4	
Use of other sedation infusions (number/patients on study; dose (SD))								
Morphine (mcg/kg/h)	CHOSEN	21/21	20/20	15/17	10/12	6/8	6/7	4/5
		49 (17)	45 (18)	40 (20)	33 (18)	36 (19)	33 (21)	40 (22)
	Control	12/21	11/20	8/14	6/11	5/6	4/4	2/3
		36 (19)	36 (21)	36 (21)	37 (24)	34 (17)	30 (18)	10 (0)
	*p* value	0.001	0.001	0.10	0.19	0.99	0.99	0.99
		0.06	0.26	0.70	0.73	0.87	0.80	0.14
Midazolam (mcg/kg/min)	CHOSEN	16/21	17/20	9/17^a^	6/12	3/8	3/7	1/5
		2.1 (0.9)	1.6 (0.7)	1.7 (0.6)	1.6 (0.9)	1.3 (0.8)	2.7 (2.1)	2
	Control	8/21	7/20	6/14	4/11	2/6	2/4	1/3
		2.3 (1.5)	2.5 (1.7)	2.6 (2.0)	3.0 (2.2)	1.5 (0.7)	1.5 (0.7)	1
	*p* value	0.03	0.003	0.72	0.68	0.99	0.99	0.99
		0.68	0.07	0.34	0.18	0.82	0.52	0.99

Data given as *n* (%), mean (SD), or median (IQR). Comparisons by Fisher’s exact and *t* test or paired *t* test, as appropriate

^a^There was a statistically significant decrease in proportion of patients on midazolam infusion between day 2 and day 3 in the CHOSEN group (*p* = 0.03 by chi-square) but not in the control group (*p* = 0.64)

### Main secondary outcomes

The number of prn doses of sedation required by patients in the CHOSEN arm decreased from 4.7 doses (SD 3.3) 12 h pre CH infusion to 2.6 doses (SD 2.8) 12 h post CH infusion (*p* = 0.009); there was no change in the historical control patients (*p* = 0.74). The time to titration to adequate sedative effect and time to awakening on discontinuation or lowering of sedation infusions did not differ between the CHOSEN and historical control groups (Table 2). The fluid balance at 48 h was − 2 (SD 45) ml/kg in the CHOSEN group vs. 26 (SD 46) ml/kg in historical controls, *p* = 0.051. The use of morphine and midazolam infusions was less on day 1 and 2 in the historical controls (vs. CHOSEN patients), but not different from days 3–7, and the dose of these infusions when used was similar (Table 3). The proportion of patients on midazolam infusion between days 2 and 3 reduced in the CHOSEN group (from 17/20 (85%) to 9/17 (53%), *p* = 0.03) but not in the historical controls (from 7/20 (35%) to 6/14 (43%), *p* = 0.64) (Table 3).

### Other secondary outcomes

Other outcomes examined are shown in Tables 2, 3 and Additional file 3: Tables S1 and S2. Ventilator hours and PICU length of stay was lower in the CHOSEN group vs. the historical controls, but not statistically significantly so (*p* = 0.12 and 0.17). No patient in either group failed extubation due to excessive sedation effect. There were no significant differences in post-extubation withdrawal measures, including signs of withdrawal, or enteral narcotic, benzodiazepine, or clonidine started within 48 h of extubation. The use of midazolam and narcotic infusions from 24 h pre extubation to 24 h post extubation significantly dropped in CHOSEN patients (from 11/21 (52%) to 2/21 (10%) on midazolam, *p* = 0.003; and from 21 (100%) to 12 (57%) on narcotic, *p* = 0.001), but not in historical controls (*p* = 0.22 and 0.12) (Additional file 3: Table S1). The use of narcotic infusion dropped further between 24 h post extubation to 48 h post extubation in CHOSEN patients (to 5/21 (24%), *p* = 0.03), but not in historical controls (*p* = 0.33) (Additional file 3: Table S1). Use of inotropes and inotrope scores did not differ between the groups. Detailed information on sedation variables are given in Additional file 3: Table S2.

## Discussion

In this study we tested a novel low-cost method of delivery of the commonly used sedative medication CH in PICU patients. The main findings of this study include the following. First, continuous enteral infusion of CH was feasible in 95% of patients, used for a mean of 4.5 (SD 2.2) days. The time to titration of adequate sedation was short (mean 1.4 (SD 1.3) days). Second, continuous enteral infusion of CH was effective, associated with a statistically significant reduction in the number of prn doses of sedation from 12 h pre baseline to 12 h post baseline, and in the use of midazolam infusions between day 2 and 3 of ventilation. This reduction did not occur in the historical controls group. The doses used for continuous CH enteral infusion on days 1–3 were usually from 5–9 mg/kg/h, providing the first dose-finding information of which we are aware. Third, continuous infusion of enteral CH was associated with a lower positive fluid balance at 48 h compared to historical controls. Other patient important outcomes were not statistically different between groups, including ventilator hours, PICU length of stay, measures of drug withdrawal after extubation, hours to awakening on weaning of sedation, and inotrope scores. There was a statistically significant decrease in the use of midazolam and narcotic infusions in the 24–48 h after extubation in the group on continuous enteral infusion of CH, but not in the historical controls. Finally, there was one possible SAE comprising a punctate discrete duodenal perforation in a 16-month-old patient with croup who had been treated with regular dexamethasone and ibuprofen for 3 days in the group on continuous enteral infusion of CH.

There are some concerns with this study that warrant discussion. First, the matching process was sub-optimal, and there were clinically significant imbalances at baseline between the CHOSEN and retrospectively identified historical control groups (such as numbers of patients with congenital heart disease, bronchiolitis, non-invasive ventilation, and medical reasons for admission). In fact, we do not suggest the use of continuous CH enteral infusion in patients without a protected airway, such as those having non-invasive ventilation. These baseline imbalances are also reflected in the statistically significantly lower use of morphine and midazolam infusions in the first and second day of the study (Table 3), and in the wide SD of ventilator hours and PICU days (Table 2) in the historical control group. We believe that these imbalances reduced the ability to detect differences in outcomes between the CHOSEN and historical control groups. For this reason, we emphasize the difference within groups in number of prn doses of sedation between 12 h pre baseline to 12 h post baseline, in use of midazolam infusion for sedation between day 2 and 3 of the study, and in use of midazolam and narcotic infusions between 24 h pre extubation to 24 h post-extubation. In addition, the lack of difference in measures of drug withdrawal post extubation between groups (Table 2) was present even though there was the imbalance in the proportion of patients on narcotic and midazolam infusion 24 h pre extubation (lower in the historical control group; Additional file 3: Table S1). Thus, the differences between the CHOSEN and historical control groups should be considered to reflect the *minimal* efficacy of the continuous enteral infusion of CH.

Second, the SAE of a punctate discrete duodenal perforation in the first part of the duodenum was considered possibly related to the continuous enteral infusion of CH (the tip of the feeding tube was at the junction of the 2nd and 3rd part of the duodenum). This is not a common complication in previously well children admitted to the PICU. The product monograph for CH lists gastric irritation as an adverse effect, and suggests CH be avoided in patients with gastritis, esophagitis, or gastric or duodenal ulcer; in overdose the product monograph states that gastric necrosis, perforation, gastrointestinal hemorrhage and esophageal stricture have also been reported [8, 18, 19]. Thus, the local research ethics board defined this SAE as being within “expected” adverse events. The patient had risk factors for a duodenal ulcer including being treated with regular dexamethasone and ibuprofen without gastrointestinal bleeding prophylaxis. A recent review suggests that proton pump inhibitors are indicated in patients taking systemic corticosteroids and concomitant non-steroidal anti-inflammatory drugs, due to the higher risk of peptic ulcers [20]. Further experience with continuous infusion of enteral CH will be necessary in order to determine whether this serious adverse event was likely attributable to the CHOSEN intervention.

There are limitations to this study. First, the small sample size comprising 21 patients in each group, all from a single center, limit the conclusions that can be made. This sample size was smaller than the calculated sample size of 32 patients that would be required to estimate feasibility as 75% with narrow 95% confidence intervals. The study was stopped before more patients were recruited due to funding constraints. The continuous enteral CH infusion was found to be feasible in 95%, with wide 95% adjusted Wald CI 76–99%. Second, the imbalance in baseline characteristics between the CHOSEN and historical control groups was discussed above. We believe that this would mean that the results of the study reflect the minimal efficacy of continuous infusion of enteral CH. Third, there was no protocol for sedation management, acceptable depth of sedation, or for scoring of the level of sedation. In addition, no serum levels or pharmacodynamic investigations were done to direct therapy. Nevertheless, this study reflects the management of sedation based upon the bedside team’s best judgement in a center that did not use sedation scoring. Fourth, there was multiple statistical testing without correction of *p* values. This problem was mitigated by pre-specifying the four main secondary outcomes as reported, with the other outcomes considered as exploratory findings in this pilot study. Finally, one might wonder why we have not implemented CH enteral infusion as standard practice in our PICU. This pilot project was considered a research study, and not a quality improvement project, and thus we wanted to await peer-reviewed publication of the results before implementing this as a standard practice (which many members of the PICU staff have requested).

There are some strengths of this study. We tested a novel, simple, and low-cost intervention with a safe drug that is commonly used for sedation in PICU. This novel method of delivery of CH by continuous enteral infusion was found to be feasible and effective. By specifying the primary and main secondary outcomes, we limited the amount of multiple statistical testing thus improving the reliability of the statistical results. Finally, this pilot study provides information that will allow for sample size estimation for future randomized studies of this intervention.

## Conclusions

Delivering CH as a continuous enteral infusion is feasible, effective, and may be associated with less positive fluid balance. This small pilot trial does not allow definitive conclusions about the relative efficacy of continuous enteral infusion of CH on other outcomes including ventilator days, PICU days, vasoactive support, or drug withdrawal, although there was no signal of any adverse effect on these outcomes. Whether there is a risk of duodenal perforation as occurred in one patient in this study requires further study.

## Additional files

Additional file 1: The study physician orders and instructions for administering enteral chloral hydrate infusion. (PDF 1548 kb)

Additional file 2: The CHOSEN study case report form. (DOCX 229 kb)

Additional file 3: Supplementary tables for the CHOSEN study results. **Table S1.** Detailed information on sedation from 24 h prior to 48 h after extubation. **Table S2.** More details of the sedation given on day 1 to 7 of study in cases and controls. (DOCX 25 kb)

## Abbreviations

CH: Chloral hydrate
IQR: Interquartile range
PELOD: Pediatric Logistic Organ Dysfunction score
PICU: Pediatric intensive care unit
PRISM: Pediatric Risk of Mortality score
prn: as required
SD: Standard deviation
