# Supplementary material for: Chloral hydrate enteral infusion for sedation in ventilated children: the CHOSEN pilot study
Source: Crit Care. 2017 Nov 26;21:290. doi: 10.1186/s13054-017-1879-7 (PMC5702481; doi:10.1186/s13054-017-1879-7)

Chloral Hydrate enteral infusion fOr Sedation in ventilated children: the CHOSEN pilot study.

Authors: Ari R Joffe MD[1], Jessica Hogan RN[2], Cathy Sheppard RN[2], Gerda Tawfik BSc Pharm[3], Jonathan P Duff MD[1], Gonzalo Garcia Guerra MD[1]

Additional File 1: The study physician orders and instructions for administering enteral chloral hydrate infusion.

Journal: Critical Care

Chloral Hydrate enteral infusion fOr SEdation in veNtilated children: The CHOSEN pilot study.

The purpose of this study is to determine whether chloral hydrate enteral infusion reduces the requirements for other sedatives by infusion and PRN doses

1. Starting the infusion:

a) Chloral Hydrate 10 mg/kg (_____ mg) NG/NJ/GT/GJ loading dose, followed by:

b) Chloral Hydrate 5 mg/kg/hr **enterally**, as an infusion, y-infused into the **feeding tube** (NG, NJ, GT, GJ) with the feeds. (mix as 50mg/ml solution...see dilution directions at bedside)

2. If the patient is agitated:

a) If the infusion is less than 9 mg/kg/hr, give chloral hydrate bolus (*please give prior to using other prn sedation, if possible*) 3mg/kg (_______ mg) NG/NJ/GT/GJ up to q1h PRN, followed by:

b) An INCREASE in the chloral hydrate infusion by 1 mg/kg/hr up to a MAXIMUM of 9 mg/kg/hr

c) If the infusion is already at maximum (9 mg/kg/hr), **no chloral hydrate PRN may be given** therefore other PRN's may be given

3. If the patient is well sedated:

a) If the patient is over-sedate for at least 4 hours and has not required any PRN sedation, please first decrease other sedation infusions being used. If the sedation is weaned to the lowest amount allowed, then decrease the chloral hydrate infusion by 1-2 mg/kg/hr up to q3h PRN.

4. Discontinuing the infusion for extubation:

a) Decrease the chloral hydrate infusion rate by half, 6 hours prior to extubation and stop the infusion 3 hours before extubation is planned.

b) If the infusion needs to be restarted:

   a. Restart the infusion at the previously running rate prior to wean

   b. If the patient is agitated – give chloral hydrate 10 mg/kg (____________________ mg) NG/NJ/GT/GJ loading dose

Physician Signature: ______________________________

Please page the research nurse on call (see research call schedule binder or white board at unit desk) for any questions you may have.

How to Set Up Your CHOSEN Enteral Chloral Infusion!

For any inquiries, contact Jessica Hogan at jessicalynn.hogan@albertahealthservices.ca, page through 780-401-0819, or contact any research member working on your shift.

1. Load the diluted chloral-primed 20 mL syringe, attached to the feeding tube, into the CHOSEN-specific pump set.

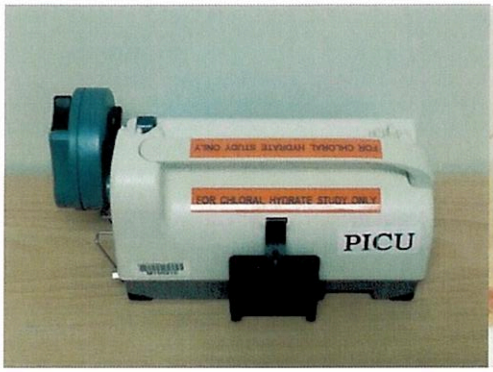

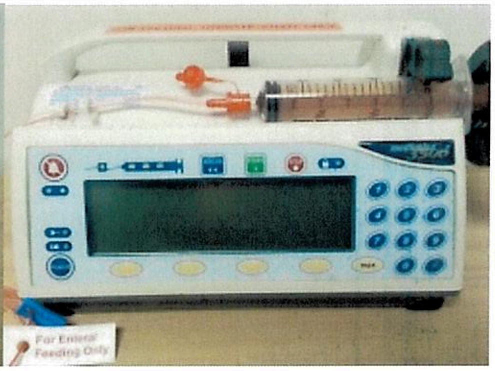

2. Turn on syringe pump and allow for self-test.

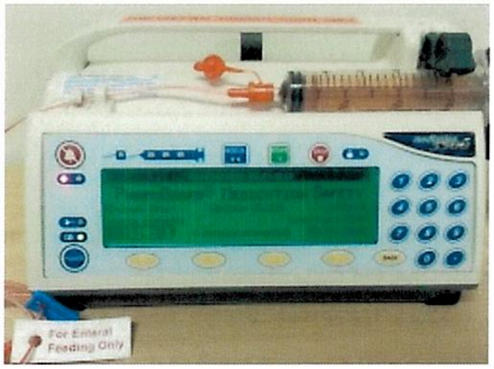

3. Press 1 for "Chloral Study".

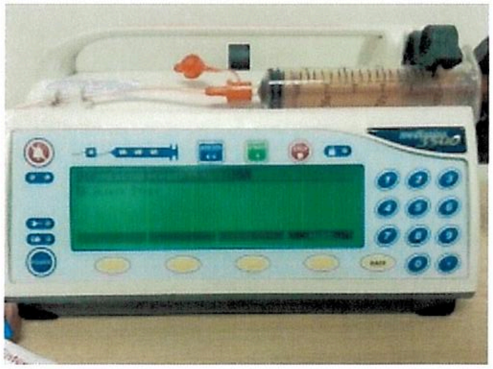

4. Press 1 for “Chloral Hydrate 50 mg/mL Enteral”.

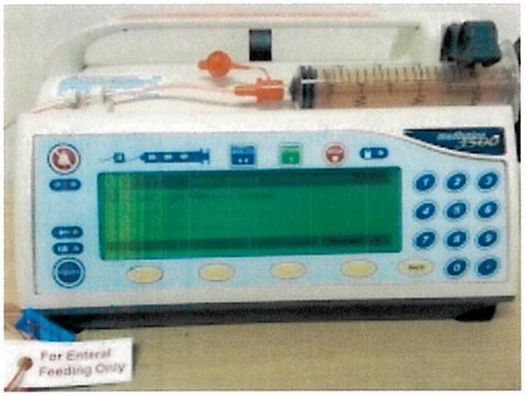

5. Press “Yes” at ***Confirm Lib Selection*** screen for “Chloral Hydr 50 mg/mL Enteral”.

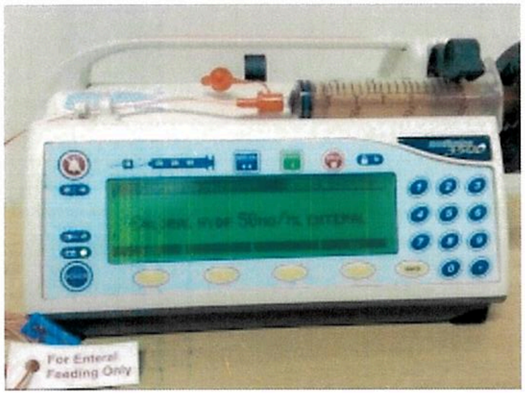

6. Press “Yes” at ***Drug Alert*** screen for “For Enteral Use Only”.

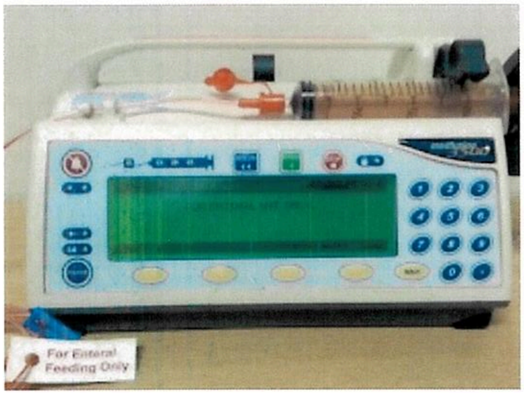

7. Press “Enter” at ***Load Syringe – Press Enter When Ready*** screen to confirm B-D 20 mL syringe selection.

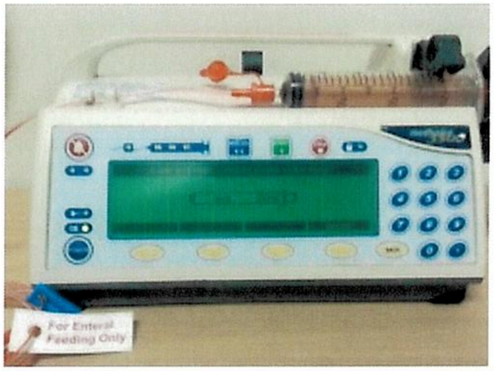

8. **THESE NEXT STEPS WILL NOT DELIVER A BOLUS TO YOUR PATIENT** (*The pump merely ensures we are setting up a standardized bolus; this can be changed at any time when you are actually delivering a 3 mg/kg bolus of chloral.*)

   a. Select appropriate weight of your patient.

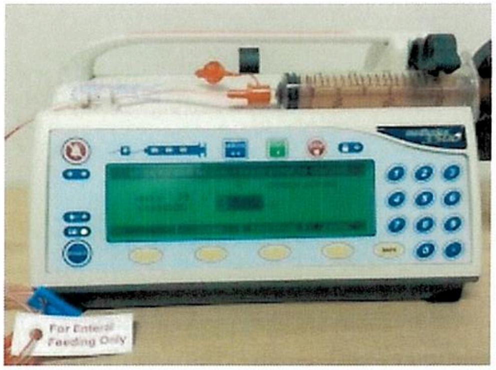

   b. Press 3 for “3 mg/kg”.

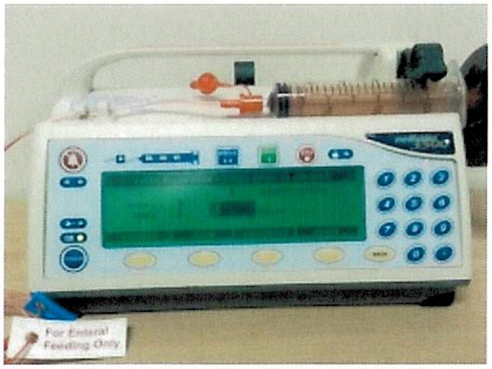

c. Press “Yes” to confirm total chloral dose. *DOUBLE CHECK with independent calculation before confirming*

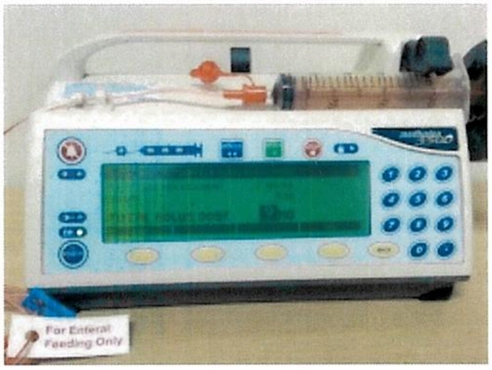

d. Enter bolus time, any time between 7 seconds and 59 seconds, for delivery. (15 seconds is a recommended standard, to mimic a manual bolusing of chloral.)

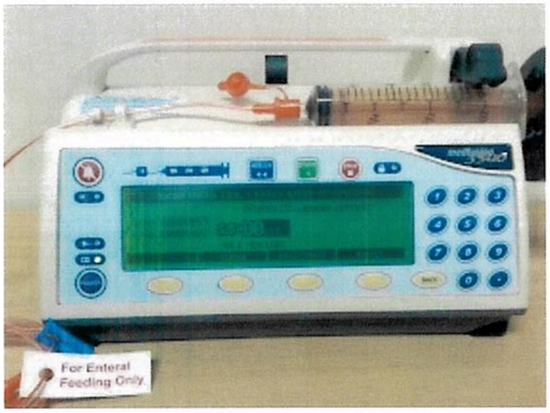

9. Prime tubing with pump, if line not yet primed. Press green “Start” button.

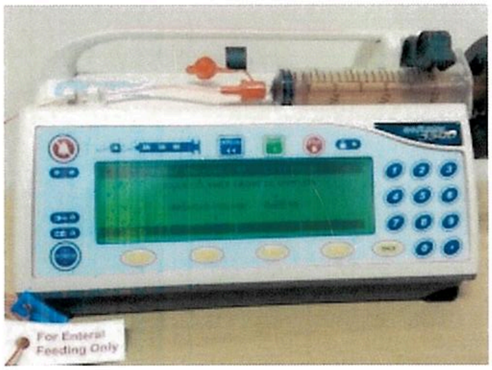

10. **REMEMBER: ALWAYS PERFORM YOUR DOUBLE-CHECK** at the final infusion set-up screen with a fellow nurse before starting your chloral infusion.

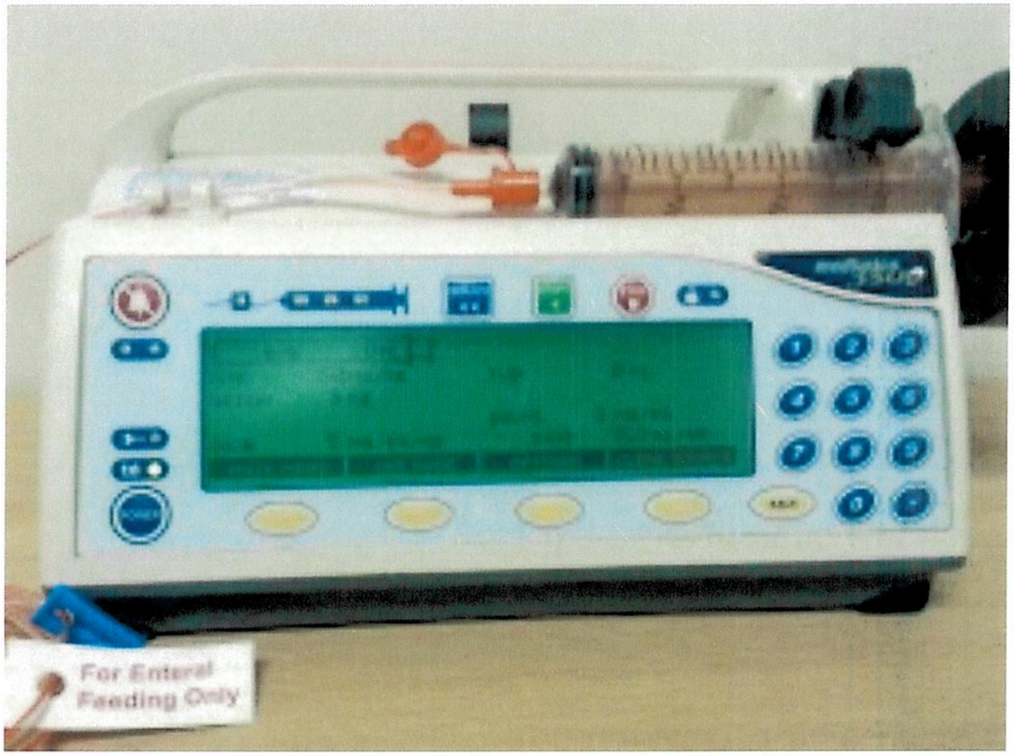

CONGRATULATIONS!

You have just started your lovely patient on an enteral chloral hydrate infusion.

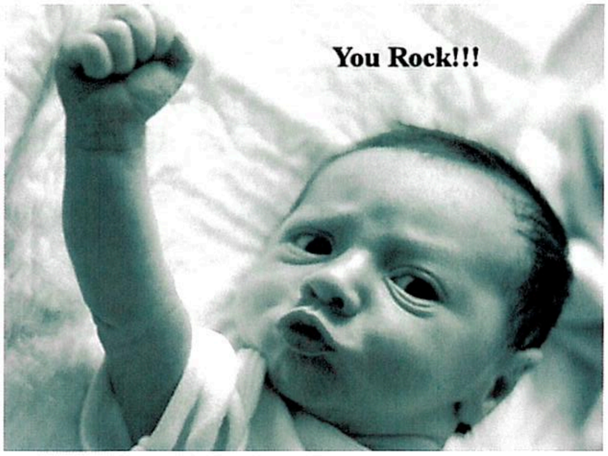

Supplement: Supplementary file 1 — The study physician orders and instructions for administering enteral chloral hydrate infusion. (PDF 1548 kb) [file 13054_2017_1879_MOESM1_ESM.pdf]
