# Supplementary material for: Chloral hydrate enteral infusion for sedation in ventilated children: the CHOSEN pilot study
Source: Crit Care. 2017 Nov 26;21:290. doi: 10.1186/s13054-017-1879-7 (PMC5702481; doi:10.1186/s13054-017-1879-7)
Supplement: Supplementary file 2 — The CHOSEN study case report form. (DOCX 229 kb) [file 13054_2017_1879_MOESM2_ESM.docx]

**Chloral Hydrate enteral infusion fOr Sedation in ventilated children: the CHOSEN pilot study.**

**Authors:** Ari R Joffe MD^1^, Jessica Hogan RN^2^, Cathy Sheppard RN^2^, Gerda Tawfik BSc Pharm^3^, Jonathan P Duff MD^1^, Gonzalo Garcia Guerra MD^1^

**Additional File 2:** The CHOSEN study case report form.

**Journal:** Critical Care

**Chloral Hydrate enteral infusion fOr SEdation in veNtilated children.**

**The CHOSEN pilot study.**

**CASE REPORT FORM**

*Please complete and return to*:

CHOSEN Project Manager

Stollery Children’s Hospital University of Alberta

Cathy Sheppard RN

Jessica Lynn Hogan RN

**Case Report Forms: Procedure Manual Notes**

**General Instructions**

· At the top of each page, enter your the **Patient’s Initials** (first, middle, last), and the patient **Number**

· Enter dates in the format dd / mm / yyyy (i.e. October 22, 1998 is 22 / Oct / 1998)

· Enter times according to the 24 hour clock in the format HH:MM (i.e. 4 pm is entered 16:00)

· Do not write in shaded areas

· If data is not applicable, not known, illegible, or incorrect, enter N/A. There should be no blank spaces. If data is missing use the letter M.

· Use only **black ink.**

**Control Patient Number:** this is the same as the patient number they are matched to.

**Source Document**

Complete the information on this page and file it in the Patient Source Document Binder.

The document is to be used as reference for patient follow-up. It may also be necessary to access the patient’s medical record in the future for auditing purposes. This information will allow for accurate case identification.

**Enrolment**

After the patient has been assessed for eligibility, if he/she meets the inclusion criteria and does not meet any of the exclusion criteria, the parents will be asked to give consent for the participation of their child in the study.

## Source Document

Once this page is completed it has to be detached from the CRF and has to be kept in a binder with all the rest of the Source Document forms.

Patient name: ________________________________

Patient’s hospital number: ________________________________

Patient study number: ________________________________

**INCLUSION/EXCLUSION CRITERIA:**

**1.1 Inclusion Criteria:**

Check “**yes**” for each inclusion criteria present.

**NOTE**: all inclusion criteria must be checked “yes” in order for the patient to be eligible for the study and to be enrolled.

**CONTROL patients**: do not have consent, so this is checked “no”.

**INCLUSION/EXCLUSION CRITERIA**

**1.1 INCLUSION CRITERIA:** (all inclusion criteria must be answered “YES” to include patient)

# YES NO

□ □ 1. Age: at least 44 weeks post-conceptual age, up to age 12 years.

□ □ 2. Admitted to the Stollery Children’s Hospital PICU for <72 hours

□ □ 3. Chloral hydrate ordered by the PICU medical team for sedation.

□ □ 4. Enteral feeding tube (NG, NJ, GT, GJT, OG, or OJ) delivering feeds at any rate.

□ □ 5. Informed consent: parent or appropriate substitute decision-maker.

**INCLUSION/EXCLUSION CRITERIA :**

**1.2 Exclusion Criteria:** Check “**no**” for each exclusion criteria NOT present. **NOTE**: all exclusion criteria must be checked “no” in order for the patient to be eligible for the study.

**INCLUSION/EXCLUSION CRITERIA**

**1.2 EXCLUSION CRITERIA (**all exclusion criteria must be answered “NO” to include patient)

**YES** **NO**

□ □ 1. Weight <3 kg.

□ □ 2. Contraindication to the use of the gastrointestinal tract to receive enteral medications. □ □ 3. Short gut syndrome.

□ □ 4. Known gastric or duodenal ulcer.

□ □ 5. Severe liver dysfunction, defined as with an INR over 2, and lactate over 2mmol/L, due to liver disease.

□ □ 6. Severe renal dysfunction, defined as requiring dialysis (peritoneal, or CVVH, or Intermittent hemodialysis) for kidney dysfunction.

□ □ 7. Allergy to chloral hydrate.

**2. BASELINE AND DEMOGRAPHICS:**

**Baseline:** the time that chloral hydrate enteral infusion is started. In the control group, baseline is the time when chloral hydrate is ordered and first given enterally to the patient.

**2-1 Age in months**: If born prior to 37 weeks gestation, give corrected age in months.

**2-2 Gender**: Check either male or female

**2-4 Some specific diagnostic categories**: septic shock- known or suspected infection, with need for vasoactive medication to support cardiac output due to the sepsis (either epinephrine, norepinephrine, dopamine, dobutamine, or milrinone).

**2-5 Severity of illness measures:**

Mechanical ventilation: ventilated using an endotracheal tube or tracheostomy.

Non-invasive ventilation: ventilated using a nasal or face mask, with CPAP, or Bilevel pressures.

Inotrope score: calculated as follows:

Dopamine mcg/kg/min + Dobutamine mcg/kg/min+ (100)(epinephrine mcg/kg/min) + (100)(norepinephrine mcg/kg/min) + (10)(milrinone mcg/kg/min)

Lactate: can be from an arterial, venous, or capillary gas. Use the gas closest to the time just before when chloral hydrate was ordered (baseline).

Creatinine: use the value closest to the time when chloral hydrate was ordered (baseline).

**2. BASELINE AND DEMOGRAPHICS**

**BASELINE: *|__|__|* / *|__|__|__|* /*|__|__|__|__|*/*|__|__|__|__|***

day month year hour (24hr clock)

**2-1. Age in months: *|__|__|__|*** *Months*

**Age category** *|__|<1yr |__| 1-5yr* *|__| 6-12yr*

**2-2. Gender:** *|__| Male |__| Female*

**2-3. Weight** *|__| kg*

**2-4. Diagnostic Category:** *|__| Cardiac surgical*

**Type of congenital heart disease:** *|__|*ASD |__|VSD |__|AVSD |__|TOF |__|TGA |__|TAPVD |__|HLHS |__|Complex Single Ventricle |__|Glenn |__|Fontan |__|Other

|__|Not CHD

**RACHS-1 Score** *|__|*

*|__| Other surgical*

*|__| Medical*

**Some specific diagnostic categories** *|__| Neurosurgical patient*

*|__| Bronchiolitis*

*|__| Trauma*

*|__| Septic shock*

**2-5. Severity of illness measures**

**Mechanical ventilation:** Yes |__| No |__|

**Non-invasive ventilation:** Yes |__| No |__|

**Inotropes:** Yes |__| No |__|

Epinephrine used Yes |__| No |__|

Norepinephrine used Yes |__| No |__|

Inotrope score ***|__|__|__|***

**Lactate:** |__|__|.|__| mmol/L

**Creatinine:** |__|__|.|__| mmol/L

**3. Physiologic Measurements at baseline.**

PRISM III Score at baseline:

Using the chart below, score the PRISM III using the worst value within the first 12 hours of baseline.

Circle the appropriate units.

-Child = 12 months - 144 months (1 - 11 years)

-Adolescent > 144 months (> 12 years).

-Glasgow coma score: use the lowest value. If the patient is sedated, or paralyzed, record the estimated Glasgow coma score *before* these events, unless there has been a CNS event that resulted in a lower GCS. For example, if the patient had a GCS of 14 before sedation and/or intubation, and at baseline the patient is still sedated or paralyzed, then give a GCS score of 14. .

-**pH, pC02, pa02**: If an arterial gas, use this for pH and PaC02. If no arterial gas, may use values from a venous gas or capillary gas for pH and PaC02, but not for Pa02. Total C02 (mmol/L) can be the bicarbonate on an arterial (or, if not arterial, then venous or capillary) gas.

**3.1 PRISM Score category at baseline:** |__| <10 |__| 10-20 |__| >20

***4. PELOD: Pediatric Logistic Organ Dysfunction Score at baseline.***

Record the PELOD score **on the baseline day**.

Use only the worst value obtained on the baseline day.

If a value is not measured, it is assumed to be either identical to the last measurement (if the physician considers that the value of the variable did not change) or normal (if the physician considers that the value of the variable is normal).

- Heart rate: do not assess during crying or iatrogenic agitation.
- Systolic blood pressure: do not assess during crying or iatrogenic agitation.
- PaO_2_/FiO_2_ ratio calculation: e.g., PaO_2_ = 89 and FiO_2_ = 45%; Therefore, PaO_2_/FiO_2_ = 89/.45 = 198. If there is no arterial or capillary (add 20mmHg to the capillary P02) gas to give the Pa02, or the patient is not on mechanical ventilation or non-invasive ventilation to give the Fi02, then assume the number is normal.

PCO_2_: may be measured from arterial or venous or capillary sample.

Mechanical ventilation: the use of mask ventilation (non-invasive ventilation) is not considered as mechanical ventilation.

Glasgow coma score: use the lowest value. If the patient is sedated, or paralyzed, record the estimated Glasgow coma score *before* these events, unless there has been a CNS event that resulted in a lower GCS. For example, if the patient had a GCS of 14 before sedation and/or intubation, and at baseline the patient is still sedated or paralyzed, then give a GCS score of 14.

- Pupillary reactions: non reactive pupils must be > 3 mm. Do not assess after iatrogenic pupillary dilatation (by eye drops).

| **Pedatric Logistic Organ Dysfunction Score (PELOD)**  **.** |  |  |  |  |  | **Baseline Day** |
| --- | --- | --- | --- | --- | --- | --- |
| **PELOD Score** | **0** | **1** | **10** | **20** | **Maximum scor**e | *__/____/ 201__* |
| **Respiratory System:**   - PaO_2_/FiO_2_ ratio - PaCO_2_ mmHg (kPa) - Mechanical ventilation | > 70 (9.3) **and**  ≤ 90 (11.7) **and**  no ventilation | Ventilation | ≤ 70 (9.3) or  > 90 (11.7) |  | **10** |  |
| **Cardiovascular System**:  Heart rate (rate/min)   - < 12 yr - ≥ 12 yr | ≤ 195  ≤ 150 |  | > 195  > 150 |  |  |  |
| **Systolic BP (mmHg**)   - 1 month - 1 month - 1 yr - 1 yr. – 12 yr. - ≥ 12 yr | **and**  > 65  > 75  > 85  > 95 |  | **or**  35 – 65  35 – 75  45 – 85  55 – 95 | < 35  < 35  < 45  < 55 | **20** |  |
| **Neurological System:**  Glasgow  Pupillary reaction | 12 – 15 **and**  both reactive | 7 – 11 | 4 – 6 **or**  both fixed | 3 | **20** |  |
| **Hepatic System:**   - ALT SGOT (UI/L) - PT or INR | < 950 **and**  >60 or < 1.4 | ≥ 950 **or**  ≤ 60 or ≥ 1.4 |  |  | **1** |  |
| **Renal System:**  Creat: μmol/L (mg/dL)   - < 7 days - (7 days – 1 yr.) - (1 yr. – 12 Yr.) - ≥ 12 yr. | < 140 (<1.59)  < 55 (<0.62)  < 100 (<1.13)  < 140 (<1.59) |  | ≥ 140 (≥1.59)  ≥ 55 (≥0.62)  ≥ 100 (≥1.13)  ≥ 140 (≥1.59) |  | **10** |  |
| **Hematological System:**  White blood cell (10^9^/L)  Platelet count (10^9^/L) | > 4.5 **and**  ≥ 35 | 1.5 – 4.4 **or**  < 35 | < 1.5 |  | **10** |  |
|  |  |  |  | **TOTAL** | **71** |  |

**5. Sedation Outcomes:** outcome variables measured from baseline:

**Sedation needs:**

**5.1 Number of prn rescue sedation doses given per day on days 1-7**. Day 1 is considered the day of baseline. This includes any dose of morphine, fentanyl, hydromorphine, ativan, midazolam, ketamine, or propofol doses given for agitation. Do not include doses given for procedures such as intubation, central line, arterial line, dressing change, chest tube, sternal opening/closure, etc. Suctioning the endotracheal tube is not considered a procedure.

On day 1, the number of prn doses is recorded as:

**12 hr prior**: the number of doses prior to baseline [starting the chloral infusion in the Infusion group; first dose of chloral in the Control group]

**12 hr post**: the number of doses after baseline [starting the chloral infusion in the Infusion group; first dose of chloral in the Control group]

**NOTE**: these hours on day 1 may overlap with day 2, and may go into day 0. That is acceptable, and will be part of how we record the data.

**Note- for 5.1 to 5.4**: Data is only recorded for the days the chloral infusion is used [and in controls, for the number of days that the matched case patient had a chloral infusion].

**5.2 Time to titration of adequate sedation effect**: defined as having <3prn rescue sedation doses (as defined above) given in a 12 hr shift [a shift is 0700 to 1900, and 1900 to 0700]. Give number of days: each shift counts as half of a day. This is time from baseline.

**5.3 Total daily dose of chloral hydrate**: in mg/kg on days 1-7 including infusion and boluses. Also write the highest infusion rate in mg/kg/hr in the second column.

**5.4 Highest infusion dose of sedation:** benzodiazepine (midazolam in mcg/kg/min), narcotic (morphine in mg/kg/hr, or fentanyl in mcg/kg/hr, or hydromorphine in mcg/kg/hr), and dexmedetomidine (in mcg/kg/hr) for day 1-7.

-That dose should be running for at least 4 hours to be considered the highest infusion dose that day.

-Day 1 is considered the day of baseline.

**5.5 Time to awakening (hr) on discontinuation/lowering of sedation infusions** in preparation for extubation. Discontinuing/lowering sedation infusions is defined as when an order is written to decrease the infusions significantly in preparation for extubation in less than 24 hours later. Awakening is defined as awake enough for extubation to occur.

**5.6 Use of propofol infusion to bridge to extubation**: propofol started at least for 6 hours in order to wean off the other sedative infusions, with a view to extubation when the propofol infusion is stopped.

**5. Sedation Needs:**

**5.1 Number of prn rescue sedation doses given per day:**

| **Day** | **Narcotic** | **Benzodiazepine** | **Ketamine** | **Propofol** | **Total** |
| --- | --- | --- | --- | --- | --- |
| Day 1 | - | - | - | - | - |
| 12 hr prior | __ __ | __ __ | __ __ | __ __ | __ __ |
| 12 hr post | __ __ | __ __ | __ __ | __ __ | __ __ |
| Day 2 | __ __ | __ __ | __ __ | __ __ | __ __ |
| Day 3 | __ __ | __ __ | __ __ | __ __ | __ __ |
| Day 4 | __ __ | __ __ | __ __ | __ __ | __ __ |
| Day 5 | __ __ | __ __ | __ __ | __ __ | __ __ |
| Day 6 | __ __ | __ __ | __ __ | __ __ | __ __ |
| Day 7 | __ __ | __ __ | __ __ | __ __ | __ __ |

**5.2 Time to titration of adequate sedation effect:** |__| **.** |__| Days or |__| Not in first 7 days

**5.3 Total daily dose of chloral hydrate in mg/kg:**

| **Day** | **Total chloral hydrate dose in mg/kg** | **Highest infusion rate of chloral in mg/kg/hr** |
| --- | --- | --- |
| Day 1 | __ __ __ |  |
| Day 2 | __ __ __ |  |
| Day 3 | __ __ __ |  |
| Day 4 | __ __ __ |  |
| Day 5 | __ __ __ |  |
| Day 6 | __ __ __ |  |
| Day 7 | __ __ __ |  |

**5.4 Highest daily infusion dose of sedation until stopping the chloral infusion:**

| **Day** | **Morphine** | **Fentanyl** | **Hydromorphine** | **Midazolam** | **Dexmedetomidine** |
| --- | --- | --- | --- | --- | --- |
| Day 1 | __ __ __ | __ __ | __ __ | __ __ | ­­__ . __ |
| Day 2 | __ __ __ | __ __ | __ __ | __ __ | __ . __ |
| Day 3 | __ __ __ | __ __ | __ __ | __ __ | __ . __ |
| Day 4 | __ __ __ | __ __ | __ __ | __ __ | __ . __ |
| Day 5 | __ __ __ | __ __ | __ __ | __ __ | __ . __ |
| Day 6 | __ __ __ | __ __ | __ __ | __ __ | __ . __ |
| Day 7 | __ __ __ | __ __ | __ __ | __ __ | __ . __ |

**5.5 Time to awakening:** ***|__|__|__|* hours**

**5.6 Use of propofol infusion bridge to extubation** Yes |__| No |__|

**5.7 Feasibility of chloral hydrate infusion**: chloral infusion continued for part of that day, without discontinuation that day by the PICU medical team attributable to a potential harm and/or lack of adequate sedation.

-Potential harms are those described below; if not one of these, then write ‘other’ and describe.

-When chloral hydrate infusion was stopped, not attributable to a potential harm and/or lack of adequate sedation, the reasons include: no need for sedation anymore, too sedate despite reducing/stopping other sedatives, other (please describe).

**6. Potential harms**: did any of the following occur during the time the patient was on chloral hydrate infusion, or duration the matched patient was on chloral infusion for control patients (from day 1 to day 7, with day 1 being the day of baseline).

**6.1 Feeding tube blockage**: defined as a blocked feeding tube that requires tube replacement.

**6.2 GI bleeding**: defined as bleeding from the gastrointestinal tract (documented by blood from the gastric tube, or melena stools), and requiring transfusion of at least 10ml/kg PRBC for the GI bleed.

**6.3 New or worsened seizures**: seizures requiring treatment with an anticonvulsant.

**6.4 Feed intolerance**: defined as a decision to hold feed for >3 hours due to feed intolerance.

**6.5 New or worsened ventricular dysrhythmias**: ventricular tachycardia, ventricular fibrillation, or frequent PVCs that require treatment with either CPR, electrical conversion, or anti-dysrhythmic drug(s).

**6.6 Failed extubation due to excessive remaining sedative effect**: re-intubation or use of non-invasive ventilation, due to excessive remaining sedative effect after extubation, and occurring within 24 hours of extubation. Excessive remaining sedative effect is defined as hypoventilation (high PC02), lung collapse (on CXR), or difficulty handling oral secretions, as the reason for re-intubation or non-invasive ventilation.

**5.7 Feasibility of chloral hydrate infusion:**

| **Day** | **Chloral infusion feasible (Y/N)** | **If No: due to harm (specify)?** | **If No: due to inadequate sedation (Y/N)?** | **Chloral hydrate infusion stopped that day for another reason (specify)?** |
| --- | --- | --- | --- | --- |
| Day 1 | ___ | ____________________ | ___ | _____________________ |
| Day 2 | ___ | ____________________ | ___ | _____________________ |
| Day 3 | ___ | ____________________ | ___ | _____________________ |
| Day 4 | ___ | ____________________ | ___ | _____________________ |
| Day 5 | ___ | ____________________ | ___ | _____________________ |
| Day 6 | ___ | ____________________ | ___ | _____________________ |
| Day 7 | ___ | ____________________ | ___ | _____________________ |

**6. Potential Harms during chloral hydrate infusion or intermittent dosing:**

**6.1 Feeding tube blockage** Yes |__| No |__|

**6.2 GI Bleeding** Yes |__| No |__|

**6.3 New or worsened seizures** Yes |__| No |__|

**6.4 Feed intolerance** Yes |__| No |__|

**6.5 New or worsened ventricular dysrhythmias** Yes |__| No |__|

**6.6 Failed extubation due to excessive remaining sedation** Yes |__| No |__|

**7. PICU Outcomes:**

**7.1 Fluid balance**: in ml/kg at 24 hr and 48 hr after baseline.

**7.2 Ventilator hours:** hours from baseline to successful extubation. Successful extubation is defined as remaining extubated for at least 24 hours. If re-intubated within 24 hours, consider the patient intubated for that entire time.

**7.3 PICU length of stay**: in days, from baseline. Any portion of a shift (0700 to 1900, and 1900 to 0700) is considered one-half of a day. One day is from 0700 to 0700 the next day.

**7.4 Highest inotrope score**: on d1-7. Day 1 is the day of baseline.

**7.5 Mortality** **during or within 2 days of stopping chloral hydrate infusion**. For those in control group, record mortality up to day 9 from baseline.

**Cause of Death**: Select the primary cause of death. Please discuss the most probable cause of death with the attending physician. You may select more than one cause.

-Brain Death

-CA: Non-resuscitatable cardiac arrest, with death after failed CPR.

-WD: Withdrawal of life support. Please record whether this was due to Multiple Organ Dysfunction Syndrome, severe brain injury, refractory respiratory failure (example: refractory ARDS, neuromuscular disease and ventilator dependent), refractory shock/cardiac failure, or other.

-WH: Withholding of escalation of therapy. Please record the reason as above.

**7.6 Decision to continue chloral hydrate infusion beyond day 7 from baseline**: did the PICU medical team decide to continue the infusion after day 7, the end of the study intervention period.

**7.7 Survival to discharge from PICU:** record whether the child survived to be discharged from PICU. Truncate this at 3 months, and if the child is still alive in PICU at that time, record “yes”.

**7. PICU OUTCOMES:**

**7.1 Fluid balance**:

**At 24 hours:**  ***|__|__|__|* ml/kg**

**At 48 hours:** ***|__|__|__|* ml/kg**

**7.2 Ventilator hours:**  ***|__|__|__|* hours**

**7.3 PICU length of stay**: ***|__|__| .*** ***|__|* days**

**7.4 Highest inotrope score**:

| **Day** | **Highest inotrope score** |
| --- | --- |
| Day 1 | __ __ |
| Day 2 | __ __ |
| Day 3 | __ __ |
| Day 4 | __ __ |
| Day 5 | __ __ |
| Day 6 | __ __ |
| Day 7 | __ __ |

**7.5 Mortality**: Yes |__| No |__|

**Cause of death:** Brain Death

CA

WD

WH

**If WD or WH: please classify reason** |__| MODS

|__| Brain Injury

|__| Respiratory

|__| Cardiac/shock

|__| Other

**7.6 Decision to continue chloral hydrate infusion:** Yes |__| No |__|

**7.7 Survival to discharge from PICU**  Yes |__| No |__|

**7.8 Drug withdrawal:** were there signs of withdrawal after extubation and discontinuation of chloral hydrate. Signs of withdrawal are according to the decision of the PICU attending team. In control patients, this is the time just before and after extubation.

**Withdrawal table:**

**Day:** the max dose and number of PRN doses are for the given 24 hour period: in the 24 hours prior to stopping the chloral infusion; in the first 24 hours after stopping the chloral infusion; and in the second 24 hours after stopping the chloral infusion. For the control group, these are the respective periods before and after extubation.

**Max infusion dose**: this is the maximum dose that was sustained for at least 6 hours during that period

**Type of narcotic**: use the abbreviation M, H, or F. M: morphine; H: hydromorphone; F: fentanyl

**Oral narcotic and/or ativan and/or clonidine started regularly**: these are given for withdrawal symptoms; and started, or increased, *anytime* during the first 48 hours after the chloral hydrate infusion is stopped.

**WD Score:** this is the maximum withdrawal score recorded on two consecutive assessments during the specified interval. For example, if the highest two consecutive scores are recorded as 10 and 7, then the maximum WD score on two consecutive assessments is 7.

These are recorded to figure out if, when withdrawal occurs,

-the patient was on higher doses of narcotic or BDZ infusion prior to stopping chloral, and/or

-on lower doses of narcotic or BDZ after stopping chloral; and/or

-not being treated for narcotic or BDZ withdrawal.

**7.8. Signs of withdrawal after discontinuation of chloral hydrate:** Yes |__| No |__|

**Withdrawal Table:**

| **Day** | **Max narcotic infusion dose (and type of narcotic)** | **Number of PRN doses of narcotic given** | **Max midazolam**  **infusion dose** | **Number of PRN doses of BDZ given** | **Max dexmedetomidine infusion dose** |
| --- | --- | --- | --- | --- | --- |
| 24 hr prior to chloral stopping | _____mcg/kg/hr of ___ | ________ | _____mcg/kg/min | ________ | _______mcg/kg/min |
| 24 hr after chloral stopped | _____mcg/kg/hr of _____ | ________ | _____mcg/kg/min | ________ | _______mcg/kg/min |
| 48 hr after chloral stopped | _____mcg/kg/hr of _____ | ________ | _____mcg/kg/min | ________ | _______mcg/kg/min |

**M: morphine; H: hydromorphone; F: fentanyl**

| **Day** | **WD score** | **Enteral narcotic started regularly** | **Enteral ativan started regularly** | **Enteral clonidine started regularly** |
| --- | --- | --- | --- | --- |
| 24 hr prior to chloral stopping | ________ | - | - | - |
| 24 hr after chloral stopped | ________ | - | - | - |
| 48 hr after chloral stopped | ________ | Y/N | Y/N | Y/N |

**8. WITHDRAWAL FROM THE STUDY:**

**WITHDRAWAL FROM THE STUDY:** Check "yes" if the patient has been withdrawn from the study during the period of chloral infusion, and give the reasons for this.

**8. WITHDRAWL FROM THE STUDY**

| **Withdrawal from study** | \|__\| yes \|__\| no |
| --- | --- |
| **If yes**, check the appropriate reason | |
| **8-1** Parents asked to withdraw the child from the trial:  (*Justification:____________________________________* ) | \|__\| yes \|__\| no |
| **8-2** Physician asked to withdraw the child from the trial:  (*Justification:________________________________* ) | \|__\| yes \|__\| no |
| **8-3** Other cause of withdrawal:  (*Specify:*_______________________________________ ) | \|__\| yes \|__\| no |
| **8-4** Date of withdrawal | *\|__\|__\| \|__\|__\|__\| \|__\|__ \|__\|__\|*  *DD M M M Y Y Y Y* |
| **8-5 Day of study when withdrawal occurred (from day 1-7)** | *\|__\|__\|* |

**9: SERIOUS ADVERSE EVENTS:**

**Serious Adverse Event Information:**

Record all serious adverse events that occurred to the patient from baseline, until 2 days after the chloral hydrate infusion was stopped, or day 9, whichever is first.

**Date and Time:** Using the most application units (i.e. minutes, hours, days) indicate the time that the adverse event began.

**Duration:** Using the most applicable units (i.e. minutes, hours, days) indicate the duration of the adverse event.

**Intervention Relationship:** Indicate with the appropriate number the degree of relationship of the intervention to the adverse event according to the scale provided. **Please review with the attending intensivist.**

**Treatment Required:** Indicate with the appropriate number the treatment required for the adverse event according to the scale provided, more than one option may be selected.

**Patient Outcome:** Indicate with the appropriate number, the outcome of the adverse event according to the scale provided.

**Date of resolution**: Record the date that the adverse event stopped, or was resolved. If the patient died, enter the date of death. **Please review with the attending intensivist.**

**Definition of a Serious Adverse Event (SAE)**

An event is considered “serious” if any of the following situations occur:

- Death
- Life-threatening: refers to an event in which the patient was, in the view of the investigator, at risk of death from the event if medical intervention had not occurred.
- Prolonged Patient In-hospitalization: if a complication prolongs a patient’s hospitalization, the event is considered “serious”.
- Resulting in Persistent or Significant Disability/Incapacity: a substantial disruption of a person’s ability to conduct normal life functions.
- Other medically important condition (specify)

**FORM 9: SERIOUS ADVERSE EVENTS:**

**Serious Adverse Event Information**

| **Description of event** | **Date**  (dd/mmm/yyyy)  **Time**  (H H) | **Duration**  **(mins, hours)** | **Study intervention relationship**  1 = none  2 = possible  3 = probable  4 = definite  5 = insufficient data | **Treatment required (enter all that apply)**  1 = none  2 = medication  3 = surgery  4 = other therapy  5 = Stop Protocol | **Patient outcome**  1 = no sequelae  2 = sequelae   1. = death 2. = ongoing | **Date of Resolution**  (dd/mmm/yyyy) |
| --- | --- | --- | --- | --- | --- | --- |
|  |  |  |  |  |  |  |
|  |  |  |  |  |  |  |
|  |  |  |  |  |  |  |
|  |  |  |  |  |  |  |
|  |  |  |  |  |  |  |
|  |  |  |  |  |  |  |
|  |  |  |  |  |  |  |
|  |  |  |  |  |  |  |

**10. SIGN OFF SHEET:**

# Sign Off Sheet

**This sheet must be completed. By signing this page the parties state that the forms have been reviewed and are deemed complete and accurate**.

**10: SIGN OFF SHEET**

Case Report Form to be signed off when the data has been checked as accurate and complete.

**Research Assistant:** ________________________________**Date:**_________________________

**Site Investigator:** ________________________________**Date:**_________________________

A COPY OF THIS CASE REPORT FORM SHOULD BE KEPT ON FILE AT YOUR INSTITUTION WITH THE SOURCE DOCUMENT SHEET. THE SOURCE DOCUMENT (PAGE 1) IS TO BE KEPT IN THE PATIENT STUDY BINDER.
