# Supplementary material for: Chloral hydrate enteral infusion for sedation in ventilated children: the CHOSEN pilot study
Source: Crit Care. 2017 Nov 26;21:290. doi: 10.1186/s13054-017-1879-7 (PMC5702481; doi:10.1186/s13054-017-1879-7)
Supplement: Supplementary file 3 — Supplementary tables for the CHOSEN study results. Table S1. Detailed information on sedation from 24 h prior to 48 h after extubation. Table S2. More details of the sedation given on day 1 to 7 of study in cases and controls. (DOCX 25 kb) [file 13054_2017_1879_MOESM3_ESM.docx]

**Chloral Hydrate enteral infusion fOr Sedation in ventilated children: the CHOSEN pilot study.**

**Authors:** Ari R Joffe MD^1^, Jessica Hogan RN^2^, Cathy Sheppard RN^2^, Gerda Tawfik BSc Pharm^3^, Jonathan P Duff MD^1^, Gonzalo Garcia Guerra MD^1^

**Additional File 3.** Supplementary tables for the CHOSEN study results.

Table S1. Detailed information on sedation from 24 hrs prior to 48 hrs after extubation.

Table S2. More details of the sedation given on day 1 to 7 of study in cases and controls.

**Journal:** Critical Care

Table S1. Detailed information on sedation from 24 hrs prior to 48 hrs after extubation.

| **Variable** | **CHOSEN (n=21)** | **Control (n=21)** | **P-value** |
| --- | --- | --- | --- |
| **24 hr pre-extubation sedation wean** | | | |
| Narcotic infusion | 19 morphine, 2 hydromorphine (100%)  Morphine: 37 (17) mcg/kg/hr | 11 morphine, 2 hydromorphine (62%)  Morphine: 34 (19) mcg/kg/hr | 0.002  0.63 |
| Doses narcotic prn | 1.1 (1.2) | 0.6 (0.9) | 0.13 |
| Midazolam infusion | 11/21 (52%)  1.5 (0.8) mcg/kg/min | 6/21 (29%)  1.3 (0.8) mcg/kg/min | 0.12  0.76 |
| Doses BDZ prn | 1.0 (1.3) | 0.5 (1.1) | 0.17 |
| Dexmedetomidine infusion | 5/21 (24%)  0.6 (0.3) mcg/kg/hr | 3/21 (14%)  0.6 (0.4) mcg/kg/hr | 0.43  0.89 |
| WD score | 1/21 (5%)  5 | 17/21 (81%)  2.1 (2.1) | <0.001  - |
| **24 hr post-extubation sedation wean** | | | |
| Narcotic infusion | Morphine 11, hydromorphine 1 (57%)  Morphine: 22 (16) mcg/kg/hr | Morphine 8, hydromorphine 1 (43%)  Morphine: 18 (18) mcg/kg/hr | 0.36  0.55 |
| p-value for proportion 24hr pre- to post-extubation | 0.001 | 0.22 |  |
| Doses narcotic prn | 1.0 (1.4) | 0.8 (1.4) | 0.66 |
| Midazolam infusion | 2/21 (10%)  1.5 (0.7) mcg/kg/min | 2/21 (10%)  1.5 (0.7) mcg/kg/min | 0.99  0.99 |
| p-value for proportion 24hr pre- to post-extubation | 0.003 | 0.12 |  |
| Doses BDZ prn | 0.5 (0.9) | 0.2 (0.7) | 0.27 |
| Dexmedetomidine infusion | 3/21 (14%)  0.4 (0.1) mcg/kg/hr | 2/21 (10%)  0.7 (0.6) | 0.63  0.46 |
| p-value for proportion 24hr pre- to post-extubation | 0.43 | 0.63 |  |
| WD score^a^ | N=12/21 (57%)  5.8 (4.0) | N=16/21 (76%)  4.2 (3.4) | 0.33  0.27 |
| **48 hr post-extubation sedation wean** | | | |
| Narcotic infusion | Morphine 5/21 (24%)  26 (11) mcg/kg/hr | Morphine 6/21 (29%)  10 (32) mcg/kg/hr | 0.73  0.03 |
| p-value for proportion 24hr post- to 48hr post-extubation | 0.03 | 0.33 |  |
| Narcotic prn doses | 1.2 (1.7) | 0.4 (0.9) | 0.054 |
| Midazolam infusion max | 1/21 (5%)  2 mcg/kg/min | 1/21 (5%)  1 mg/kg/min | 0.99  - |
| p-value for proportion 24hr post- to 48hr post-extubation | 0.55 | 0.55 |  |
| BDZ prn doses | 0.3 (0.9) | 0 (0) | 0.11 |
| Dexmedetomidine max | 2/21 (10%)  0.5 (0.4) | 0  - | 0.15  - |
| p-value for proportion 24hr post- to 48hr post-extubation | 0.63 | 0.15 |  |
| WD score | N=12/21 (57%)  4.3 (3.3)  4 [1.3, 6.8] | N=14/21 (67%)  2.3 (2.5)  1 [0, 5.4] | 0.75  0.10 |

Data given as n (%), or mean (SD), or median [IQR]. Comparisons by Fisher’s Exact and t-test, as appropriate. BDZ: benzodiazepine; WD: withdrawal. a. The withdrawal score used is the Withdrawal Assessment Tool-1 (Franck LS, Harris SK, Soetenga DJ, et al: The Withdrawal Assessment Tool-1 (WAT-1): An assessment instrument for monitoring opioid and benzodiazepine withdrawal symptoms in pediatric patients. Pediatr Crit Care Med 2008; 9:573–580), where a score of ≥3 may be associated with withdrawal.

Table S2. More details of the sedation given on day 1 to 7 of study in cases and controls.

| **Variable** | **Group** | **12hr pre** | **12hr post** | **day 1** | **day 2`** | **day 3** | **day 4** | **day 5** | **day 6** | **day 7** |
| --- | --- | --- | --- | --- | --- | --- | --- | --- | --- | --- |
| **PRN doses of sedation given [number of doses (SD)]** | | | | | | | | | | |
| Narcotics | CHOSEN | N=21  2.1 (2.0) | N=21  0.6 (0.8) | - | N=20  1.0 (1.7) | N=17  1.5 (2.2) | N=12  0.8 (1.3) | N=8  2.1 (1.6) | N=7  1.7 (1.3) | N=5  1.6 (1.5) |
|  | Control | N=21  1.2 (1.7) | N=21  1.3 (2.0) | - | N=20  1.0 (1.6) | N=14  1.2 (1.7) | N=10  1.3 (1.9) | N=6  1.0 (1.3) | N=4  1.8 (1.5) | N=3  1.3 (1.5) |
|  | P-value | 0.10 | 0.12 |  | 0.99 | 0.73 | 0.51 | 0.17 | 0.97 | 0.82 |
| Benzodiazepines | CHOSEN | 1.6 (2.0) | 0.9 (1.5) | - | 1.1 (1.4) | 1.6 (2.7) | 1.1 (1.4) | 2.3 (1.9) | 2.3 (2.8) | 1.2 (0.8) |
|  | Control | 0.5 (1.0) | 0.7 (1.2) | - | 0.9 (1.4) | 0.7 (1.9) | 0.3 (0.5) | 0.2 (0.4) | 0.3 (0.5) | 0 (0) |
|  | P-value | 0.04 | 0.74 |  | 0.67 | 0.31 | 0.10 | 0.02 | 0.11 | 0.03 |
| Ketamine | CHOSEN | 0.8 (1.2) | 0.4 (1.0) | - | 0.5 (1.1) | 0.1 (0.5) | 0.3 (0.6) | 0.1 (0.4) | 0.3 (0.5) | 0.4 (0.9) |
|  | Control | 1.1 (2.5) | 0.7 (2.4) | - | 0.3 (0.9) | 0.3 (0.6) | 0.7 (1.6) | 0.2 (0.4) | 0 (0) | 0 (0) |
|  | P-value | 0.59 | 0.62 |  | 0.45 | 0.40 | 0.37 | 0.84 | 0.17 | 0.48 |
| Propofol | CHOSEN | 0.1 (0.7) | 0.8 (2.3) | - | 0.9 (2.1) | 0.4 (1.1) | 0.4 (1.0) | 0 (0) | 0.4 (1.1) | 1.6 (2.6) |
|  | Control | 0.05 (0.2) | 0.7 (2.1) | - | 0.3 (0.7) | 0.1 (0.5) | 0.7 (1.6) | 0.5 (1.2) | 0.3 (0.5) | 0 (0) |
|  | P-value | 0.53 | 0.84 |  | 0.21 | 0.51 | 0.62 | 0.36 | 0.78 | 0.34 |
| Total | CHOSEN | 4.7 (3.3) | 2.6 (2.8) |  | 3.5 (3.5) | 3.5 (4.7) | 2.6 (2.2) | 4.5 (3.3) | 4.7 (4.8) | 4.8 (3.9) |
|  | Control | 2.9 (3.9) | 3.4 (5.0) |  | 2.4 (2.9) | 2.4 (3.5) | 3.0 (3.1) | 1.8 (1.7) | 2.3 (1.5) | 1.0 (1.0) |
|  | P-value | 0.12 | 0.55 |  | 0.29 | 0.45 | 0.72 | 0.10 | 0.35 | 0.16 |
| **Use of other sedation infusions [number/patients on study; dose (SD)]** | | | | | | | | | | |
| Morphine (mcg/kg/hr) | CHOSEN | - | - | 21/21  49 (17) | 20/20  45 (18) | 15/17  40 (20) | 10/12  33 (18) | 6/8  36 (19) | 6/7  33 (21) | 4/5  40 (22) |
|  | Control | - | - | 12/21  36 (19) | 11/20  36 (21) | 8/14  36 (21) | 6/11  37 (24) | 5/6  34 (17) | 4/4  30 (18) | 2/3  10 (0) |
|  | P-value | - | - | 0.001  0.06 | 0.001  0.26 | 0.10  0.70 | 0.19  0.73 | 0.99  0.87 | 0.99  0.80 | 0.99  0.14 |
| Hydromorphone (mcg/kg/hr) | CHOSEN | - | - | 0/21  - | 1/20  10 | 2/17  8 (6) | 2/12  8 (6) | 1/8  8 | 1/7  5 | 1/5  1 |
|  | Control | - | - | 2/21  7 (8) | 2/20  7 (8) | 2/14  7 (8) | 2/11  7 (9) | 0/6  - | 0/4  - | 0/3  - |
|  | P-value | - | - | 0.49  - | 0.99  0.82 | 0.99  0.95 | 0.99  0.93 | 0.99  - | 0.99  - | 0.99  - |
| Midazolam (mcg/kg/min) | CHOSEN | - | - | 16/21  2.1 (0.9) | 17/20  1.6 (0.7) | 9/17^a^  1.7 (0.6) | 6/12  1.6 (0.9) | 3/8  1.3 (0.8) | 3/7  2.7 (2.1) | 1/5  2 |
|  | Control | - | - | 8/21  2.3 (1.5) | 7 /20  2.5 (1.7) | 6/14  2.6 (2.0) | 4/11  3.0 (2.2) | 2/6  1.5 (0.7) | 2/4  1.5 (0.7) | 1/3  1 |
|  | P-value | - | - | 0.03  0.68 | 0.003  0.07 | 0.72  0.34 | 0.68  0.18 | 0.99  0.82 | 0.99  0.52 | 0.99  0.99 |
| Dexmedetomidine  (mcg/kg/hr) | CHOSEN | - | - | 3/21  0.6 (0.2) | 3/20  0.5 (0.1) | 4/17  0.5 (0.2) | 3/12  0.5 (0.2) | 3/8  0.4 (0.1) | 3/7  0.6 (0.2) | 2/5  0.7 (0) |
|  | Control | - | - | 2/21  0.8 (0.1) | 3/20  0.6 (0.2) | 3/14  0.6 (0.2) | 3/11  0.6 (0.2) | 2/6  0.5 (0.1) | 2/4  0.5 (0.1) | 0/3  - |
|  | P-value | - | - | 0.99  0.33 | 0.99  0.25 | 0.99  0.41 | 0.99  0.57 | 0.99  0.41 | 0.99  0.66 | 0.46  - |

Data given as n (%), or mean (SD). Comparisons by Fisher’s Exact and t-test, as appropriate.

a. There was a statistically significant decrease in proportion of patients on midazolam infusion between day 2 and day 3 in the CHOSEN group [p=0.03 by Chi-square] but not in the control group [p= 0.64].
